# Use of Alcohol

**Published:** 1890-06-21

**Authors:** 


### DIETETICS.

### USE OF ALCOHOL.

"Alcohol from a Medical Point of View" is the title of an article by Dr. John Johnson in the *Pro. Med. Journal.* First Dr. Johnson asks, "Is alcohol a food, and does it supply strength to the body?" In reply, he asserts that alcohol is in no sense a food. Instead of helping work it really hinders it. This, Dr. Johnson states, has been proved over and over again by observing its effects on large bodies of workmen, on armies, &c., the results invariably showing that more and better work is done without alcohol than with it. The march across the African desert, the last English expedition to the North Pole, the experience of Captain Webb, of our best cricketers, and the orders of trainers to their pupils are referred to as examples. Also, Dr. Johnson says, that although alcohol may be in some degree regarded as a food economiser, this is an argument against its use. The elimination of waste tissues from the body is diminished by alcohol. But it must not be forgotten that accumulation of waste products within the tissues is in reality a proof of disease. It is well known that those accustomed to take much drink are "bad subjects" when injured or attacked by acute disease.

The next query Dr. Johnson proposes and answers is, "Is alcohol a stimulant?" He says that alcohol acts by paralysing the regulating and controlling the nerves of the heart, thus making that organ beat faster. Hence the flushing of the skin and the rapid action of the heart caused by alcohol. In calling alcohol a stimulant we are wrong, for it is a powerful sedative.

Thirdly, the author asks, "Does alcohol assist digestion?" Here Sir William Roberts is quoted, who wrote, "It was found that no quantity of alcohol ever increased the rapidity of digestion." And Dr. Johnson adds, "It is true that if a man eats more than he ought to do, alcohol will remove the unpleasant symptoms, but this is due not to a quickening of digestion, but to a benumbing effect on the nerves of the stomach."

Then the author asks, "Does alcohol warm the body?" Most people think that a glass of spirits will "keep out the cold." This Dr. Johnson states is a mistaken notion. Alcohol really cools the body, by sending the blood which retains the heat from the internal organs to the skin, where it is most exposed and rapidly cooled. This congestion of the skin also tends to induce perspiration, the evaporation of which chills the surface, and increases the liability to take cold.

Some lengthy remarks on "alcohol in disease" conclude the paper. The author admits that alcohol is valuable in disease, but he asks, "What good can we expect from alcohol as a medicine, if we use it every day as a beverage?" But the treatment of fever by alcohol is now a thing of the past, resulting both in a diminished mortality and in a lessened expenditure. The author admits there are cases in which alcohol may be used advantageously as a medicine, and, personally, he "should not like to be deprived of the use of such a powerful and valuable drug."

Now, we are all aware that anything beyond a limited consumption of alcohol will be deleterious. But, on the other hand, we cannot advise teetotalism for the masses, who have well-balanced minds, and who, therefore, are able to avoid excess. There are conditions in which alcohol is decidedly beneficial. Notwithstanding what Sir W. Roberts says, the topical effect of alcohol on the stomach, and therefore on digestion, differs according to whether the dose is large or small. Everyday experience demonstrates that a small dose will increase the secretion of the gastric juice. Alcohol is certainly very serviceable in the prostration arising from acute illness, when, in common with other functions, digestion is much depressed. Strength doubtless is best supported by food, but at the critical time of convalescence small doses of alcohol will increase the digestive power. In the diarrhœa and vomiting, with prostration, of children, small quantities of alcohol are often most beneficial. Then, after fatigue, persons are frequently unable to take the very necessary food, but a small dose of spirits will cause the appetite to return. Ringer says, "Many dwellers in towns who lead a sedentary life, and suffer from weak digestion, find that only by the aid of alcohol in some form or other can they properly digest their food." Spirits or wine are the best remedies when the heart is suddenly enfeebled from fright, loss of blood, accidents, and other causes.

There is no doubt that alcohol if taken in larger quantities than can be assimilated renders the system prone to disease. From experiments it would appear that the body of a strong, healthy man in a temperate climate is capable of appropriating two ounces of alcohol as a maximum in the twenty-four hours. If more than two ounces is taken it may be chemically detected in the urine and breath. Approximately, two ounces of average brandy contains one ounce of alcohol; of sherry about eight ounces, of champagne fourteen ounces, of claret sixteen ounces, of bottled beer eighteen ounces, contain one ounce of alcohol. It will therefore be understood that the limits of *appropriation* of alcohol by the system may be reached by very moderate indulgence. And there is every reason to believe that the limit of *benefit* to the system is arrived at before the limit of *appropriation.* But liquors, wine, and beer are made from many things, and many samples sold contain principles as deleterious as alcohol. When, some years ago, the duty on malt was raised, a grumbler who had to pay more for his beer, inquired why this should be the case, seeing that malt did not enter much into the composition of his favourite beverage! Physiological science and experience alike teach us that a condition of system most favourable to the development of zymotic poisons is set up by the presence in the blood of organic matters in a state of change, decomposition, or fermentation. Hence the blood of the intemperate, charged with alcohol or its results, is in the condition *par excellence* favourable to the access of various maladies. The liver is also liable to become affected from indulgence in spirituous liquors. Neither does the heart escape. One ounce of alcohol raises the pulse three beats per minute, or, in other words, causes the heart to beat while its effects last at the rate of 4,300 beats more than natural in the twenty-four hours. The heart cannot be made to do this extra work without suffering. The bad effects of alcohol on the brain and nervous system are equally demonstrable. Notwithstanding, however, all that may be said against alcohol, as shown above, there are times when it is positively serviceable and beneficial. Neither do we advocate teetotalism. But we would desire to impress upon those using alcohol that it can only be taken with a view to its tonic effects, and not for the temporary stimulation it affords. Those not recollecting this, those not appreciating this, and those, although recollecting and perhaps appreciating the advice, who are too weak to act upon it, had better become teetotallers.

